# Coexisting Type 1 Diabetes, Persistent Symptoms, and Financial Issues Associate With Poorer Adherence to a Gluten-Free Diet in Celiac Disease After Transition From Pediatrics to Adult Care

**DOI:** 10.3389/fnut.2022.883220

**Published:** 2022-05-26

**Authors:** Laura Kivelä, Anna Eurén, Marleena Repo, Heini Huhtala, Katri Kaukinen, Kalle Kurppa

**Affiliations:** ^1^Celiac Disease Research Center, Tampere University, Tampere, Finland; ^2^Tampere Centre for Child, Adolescent and Maternal Health Research, Tampere University and Department of Pediatrics, Tampere University Hospital, Tampere, Finland; ^3^Children's Hospital and Pediatric Research Center, University of Helsinki and Helsinki University Hospital, Helsinki, Finland; ^4^Faculty of Social Sciences, Tampere University, Tampere, Finland; ^5^Department of Internal Medicine, Tampere University Hospital, Tampere, Finland; ^6^The University Consortium of Seinäjoki and Department of Pediatrics, Seinäjoki Central Hospital, Seinäjoki, Finland

**Keywords:** celiac disease, type 1 diabetes, gluten-free diet, transition of care, comorbidity, follow-up

## Abstract

**Purpose:**

We evaluated adherence to a gluten-free diet and associated factors in adult celiac disease patients diagnosed in childhood.

**Methods:**

Comprehensive medical data on 955 pediatric celiac disease patients was collected and study questionnaires sent to 559 who were now adults. All variables were compared between strictly adherent and non-adherent patients.

**Results:**

Altogether 237 adults (median age 27 years, 69% women) responded to the questionnaires a median of 18 (range 3–51) years after the childhood diagnosis. Altogether 78% were reportedly adherent and 22% non-adherent. The non-adherent patients had more concomitant type 1 diabetes (18% vs. 4%, p = 0.003), whereas the groups did not differ in demographic data or clinical and histological features at diagnosis, or in short-term dietary adherence. In adulthood, non-adherent patients found gluten-free diet more challenging (39% vs. 17%, *p* < 0.001) and had higher prevalence (39% vs. 19%, *p* = 0.004) and severity of symptoms. The main motivation factors for dietary adherence were attempts to avoid symptoms and complications, but these were considered less important and price of gluten-free products more important among non-adherent patients. Adherent and non-adherent patients did not differ in socioeconomic or lifestyle factors, comorbidities other than type 1 diabetes, self-reported general health, health concerns, follow-up, or in quality of life.

**Conclusion:**

Most originally pediatric celiac disease patients reported strict dietary adherence in adulthood. However, particularly those with concomitant type 1 diabetes, persistent symptoms or financial issues may require attention during the transition from pediatric to adult care.

## Introduction

Celiac disease is one of the most common food-related chronic health conditions, affecting up to 1–3% of people worldwide (1). The only currently available treatment is a life-long gluten-free diet, entailing strict avoidance of wheat, barley, rye, and other dietary products containing added gluten (2). Initiation of the diet usually results in rapid clinical and histologic recovery, whereas continuous gluten exposure predisposes to persistent symptoms and various complications (3–6). Notwithstanding the benefits, the extensive dietary restriction increases the risk for unbalanced diet and nutritional deficiencies, and may also cause financial and social burden and have a negative effect on quality of life (7–11). These challenges may lead to dietary lapses, especially in subjects not experiencing symptoms after gluten exposure (12).

Patients diagnosed in childhood comprise a special group in this context. Parents and other adults have usually taken care of their gluten-free diet until adolescence and, in spite of having lived with the diet for many years, patients themselves may be uncertain about the original reason for their diagnosis. Accordingly, adolescents have comprised a particular risk group for difficulties with the diet and non-adherence (13–17). However, data on long-term dietary adherence and associated factors after the transition from pediatric to adult care remains limited (12, 18–20). This information would be especially important to improve treatment outcomes by focusing intensified follow-up and support on those in need (12).

Our aim was to evaluate the above-mentioned issues by comparing large well-defined cohorts of long-term adherent and non-adherent adults diagnosed in childhood with celiac disease.

## Materials and Methods

### Patients and Study Design

The study was conducted in Tampere University and Tampere University Hospital. Altogether 955 children diagnosed with celiac disease in the period 1966–2014 were identified from participation in previous celiac disease studies or by a diagnostic code search from the patient records of Tampere University Hospital (21). Comprehensive medical data was collected retrospectively from the medical records. In some of the patients, data were supplemented with personal interviews. Altogether 559 patients were ≥18 years of age in September 2016, had a biopsy-proven childhood diagnosis of celiac disease and were invited to complete specific study questionnaires.

### Clinical Features at Childhood Diagnosis

Comprehensive clinical, serological, and histological data at the time of childhood diagnosis of celiac disease were collected as available.

Based on the main reason for suspected celiac disease, clinical presentation was further classified as “gastrointestinal”, “extraintestinal”, or “screen-detected”. Gastrointestinal presentation included those suffering, for example, from diarrhea, abdominal pain, or vomiting, whereas extra-intestinal complains were defined to include e.g., skin and joint symptoms, growth disturbances, elevated liver enzymes, and anemia. Reasons for risk-group screening were e.g., type 1 diabetes (T1D) and family history of celiac disease.

Growth was classified as “normal” or “poor” by applying national growth charts with age- and sex-specific reference values and information on parental height (22). Body mass index (BMI) was calculated as kg/m^2^. Anemia was defined as blood hemoglobin below the age- and sex-specific reference.

Serum transglutaminase 2 antibodies (TGA) were measured before 2011 by conventional enzyme-linked immunosorbent assay (Phadia AB, Uppsala, Sweden), and thereafter by automatized enzyme fluoroimmunoassay (Phadia). Values above 7 U/L were considered positive and the highest reported value was 120 U/L. Serum endomysial antibodies (EmA) were measured by indirect immunofluorescence using human umbilical cord as a substrate and diluted from 1:5 until 1:4000 or negative. Histological findings in small-bowel mucosa were evaluated by hospital pathologists from at least four representative and well-orientated duodenal biopsies. Degree of villous atrophy was further classified as “partial”, “subtotal”, or “total” corresponding approximately to the Marsh-Oberhuber classification IIIa-c (23).

### Follow-Up in Childhood

All children diagnosed with celiac disease routinely receive dietary counseling by a dietician. Data on attendance at follow-up visits, short-term adherence to a gluten-free diet, and treatment response were collected from patient records 6–24 months after the diagnosis. The reported adherence was further classified as “strict”, “occasional lapses”, or “no diet”. Treatment response was classified as “response” or “no response” based on alleviation of symptoms and disappearance/decrease of serum autoantibodies.

### Long-Term Health and Treatment Outcomes in Adulthood

A structured study questionnaire was used to collect data on current demographics, membership of a celiac society, family history of celiac disease, presence of one or more offspring, lifestyle, such as smoking and physical activity, possible medications, height and weight, and presence of concomitant diseases and/or celiac disease-associated complications. Patients were also asked about their general health, health concerns, restrictions in daily life due to a gluten-free diet, difficulties and strictness of the diet, possible ongoing symptoms, and current follow-up.

General health was classified as “excellent/good” or “moderate/poor”, health-related concerns as “none/minor” or “moderate/severe” and participation in follow-up as “regular” or “none/occasional”. Patients were asked to define the frequency of their difficulties with a gluten-free diet and it was classified as “none/seldom” or “sometimes/often” and that of dietary lapses as “occasional”, “1–5 per month”, “every week”, and “no diet”. For the study comparisons, all patients reporting even occasional current lapses were classified as “long-term non-adherent” and other patients as “long-term adherent”. Patients were asked whether trying to avoid symptoms and/or complications, and the price and availability of gluten-free products affected their dietary adherence. They moreover had an opportunity to describe their present and past reasons for non-adherence.

The widely used Psychological General Well-Being questionnaire (PGWB) was used to evaluate current health-related quality of life (24). It consists of 22 questions assessing six subdimensions: anxiety, depression, positive well-being, self-control, general health, and vitality. The total score is calculated as a sum of all scores, which ranges 22–132, and the sub-scores are calculated as sums of 2-4 related questions. Higher scores denote better psychological well-being.

Presence and severity of possible current gastrointestinal symptoms were evaluated with the 15 questions of the Gastrointestinal Symptom Rating Scale (GSRS) (25). The total score, calculated as a mean of all questions ranges 1–7, and sub-scores for abdominal pain, indigestion, diarrhea, constipation, and reflux are calculated as means of the related questions. Higher scores indicate more severe symptoms.

### Ethical Aspects

The study protocol was approved by the Regional Ethics Committee of Tampere University Hospital (license number R16091, 31 May 2016). All patients and/or their parents responding to the study questionnaires and/or participating in earlier interviews gave written informed consent. The ethical guidelines of the Declaration of Helsinki were followed.

### Statistics

Categorized values are reported as numbers and percentages, and numerical values with medians and quartiles. Comparison between the groups was conducted with Chi square test, Fisher's exact test, and Mann-Whitney U test as appropriate. Logistic and linear regression analyses were used to adjust for the difference between the groups in the presence of T1D. *P*-value < 0.05 was considered statistically significant. Statistical analyses were performed with SPSS v 24.0 (IBM Corp. Armonk, NY).

## Results

In total, 237 (42%) currently adult celiac disease patients responded to the questionnaires and comprised the final cohort for the study. Based on patient record data, the responders were more often women (69% vs. 52%, *p* < 0.001) and relatives of celiac disease patients (56% vs. 44%, *p* = 0.035) and had less often concomitant T1D (9% vs. 16%, *p* = 0.029) than the non-responders (*n* = 322). The groups did not differ in other clinical and histological features at diagnosis (data not shown) or short-term adherence to a gluten-free diet after 6–24 months (strict diet 86% vs. 85%, *p* = 0.228).

Altogether 186 (79%) of the 237 celiac disease patients diagnosed in childhood reported strict long-term adherence to a gluten-free diet after a median of 18 (range 3–51) years in adulthood. Fifty-one (22%) patients reported dietary lapses and, in detailed analysis, 31 (13%) of them reported occasional lapses, 10 (4%) 1–5 lapses per month, four (2%) weekly lapses and five (2%) did not adhere to the diet at all.

Coexisting T1D at childhood diagnosis was more common in long-term non-adherent than adherent patients (18% vs. 4%, *p* = 0.003). Adherent and non-adherent patients did not differ in demographic, clinical, and histological data (Table 1), in the nature and severity of symptoms (data not shown), or in the median values of hemoglobin (123 vs. 123 g/l, *p* = 0.920) and serum autoantibodies (TGA 86 U/l vs. 71 U/l, *p* = 0.393; EmA 1:500 vs. 1:500, *p* = 0.912) at diagnosis. The groups also demonstrated comparable short-term dietary adherence (Table 1), frequency of treatment response (99% vs. 100%, *p* = 1.000), and presence of follow-up (91% vs. 95%, *p* = 0.534) 6–24 months after the diagnosis in childhood.

**Table 1:** Characteristics at celiac disease diagnosis in childhood among 237 adult patients currently adherent or non-adherent to a gluten-free diet (GFD).

	**Adherent**, ***n*** **=** **186**		**Non-adherent**, ***n*** **=** **51**		
	**Median**	**Quartiles**	**Median**	**Quartiles**	**P-value**
Age at diagnosis, years	9.8	5.6, 13.8	8.9	3.6, 12.3	0.120
Year of diagnosis	1999	1986, 2004	1995	1984, 2002	0.145
Body mass index, kg/m^2^	16.6	15.1, 18.5	16.4	15.8, 18.1	0.986
	**n**	**%**	**n**	**%**	
Girls	131	70.4	33	64.7	0.433
Clinical presentation					0.558
Screen-detected^a^	35	18.9	13	25.5	
Gastrointestinal	99	53.5	24	47.1	
Extra-intestinal	51	27.6	14	27.5	
Any symptoms	135	77.1	37	75.5	0.811
Anemia	55	31.3	8	17.4	0.063
Poor growth	81	47.6	16	34.0	0.097
Degree of villous atrophy					0.405
Partial	49	29.3	18	38.1	
Subtotal	69	41.3	15	32.6	
Total	49	29.3	13	28.3	
Childhood GFD adherence^b^					0.258
Strict diet	134	87.6	33	80.5	
Occasional lapses	18	11.8	7	17.1	
No diet	1	0.7	1	2.4	

a *Due to family history of celiac disease (n = 35), previous type 1 diabetes (n = 12) or participation in research project (n=1);*

b *6–24 months after the celiac disease diagnosis. Data was available for ≥90% of cases in each variable except body mass index 160/237 and GFD adherence 194/237*.

**Figure 1:**
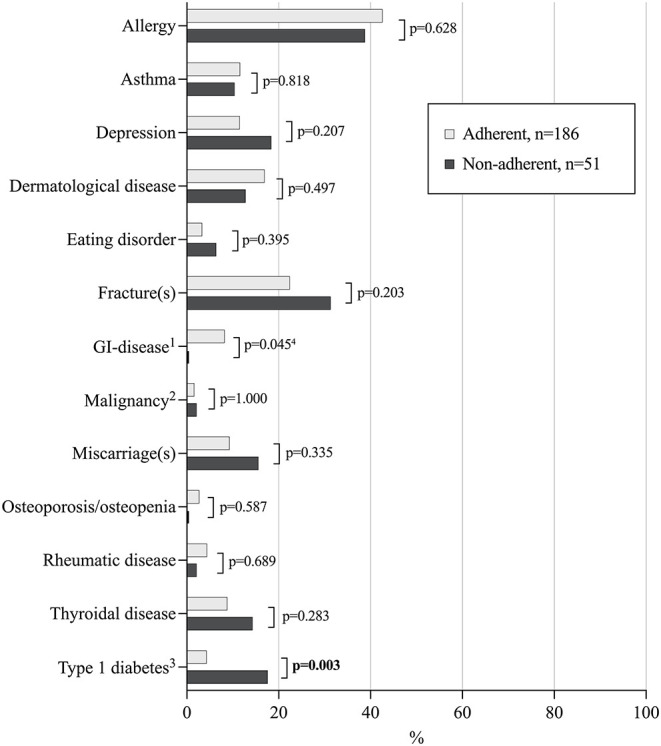
Comorbidities and possible complications in 237 adult celiac disease patients diagnosed in childhood and currently adherent or non-adherent to a gluten-free diet. ^1^E.g. inflammatory bowel disease, gastric ulcer, and irritable bowel syndrome; ^2^E.g. breast cancer, Hodgkin's lymphoma, and cancer of the central nervous system; ^3^All patients with type 1 diabetes were diagnosed prior to or concomitantly with celiac disease in childhood. ^4^Not significant after adjusting for concomitant type 1 diabetes. Statistically significant P-values presented in bold face.

Long-term non-adherent patients reported more coexisting T1D, also in adulthood (Figure 1), and all the diagnoses had been set in childhood, either before or concomitantly with celiac disease. Gastrointestinal comorbidities were less common than in adherent patients, but the difference was not significant after adjusting for groups with concomitant T1D. The groups were comparable in other comorbidities in adulthood (Figure 1). Non-adherent patients reported higher median BMI, but this difference was likewise not significant after adjustment for T1D (Table 2). The groups did not differ in current age, time from diagnosis, working/studying status, membership of a celiac society, lifestyle, presence of offspring, and family risk for celiac disease (Table 2).

**Table 2:** Sociodemographic and lifestyle characteristics in 237 adult celiac disease patients diagnosed in childhood and currently adherent or non-adherent to a gluten-free diet.

	**Adherent**, ***n*** **=** **186**		**Non-adherent**, ***n*** **=** **51**		
	**Median**	**Quartiles**	**Median**	**Quartiles**	**P-value**
Age, years	26.8	22.1, 36.3	28.5	21.5, 38.8	0.606
Time from diagnosis, years	17.7	12.2, 30.6	21.6	14.5, 32.4	0.147
Body mass index, kg/m^2^	23.2	21.2, 26.4	25.5	22.9, 26.9	0.013^d^
	***n***	**%**	***n***	**%**	
Working full- or part-time	112	78.9	36	78.3	0.930
Student	65	34.9	13	25.5	0.241
Member of celiac society	99	54.1	23	46.0	0.310
Celiac disease in the family^a^	81	44.5	22	46.8	0.777
One or more offspring	81	44.0	19	38.8	0.510
Current or earlier smoking	56	30.3	18	36.7	0.387
Use of vitamin D supplement	76	46.3	16	37.2	0.283
Prescription medication^b^	83	44.6	17	33.3	0.351
Use of gluten-free oats	172	94.0	48	98.0	0.469
Regular physical exercise^c^	155	84.2	38	77.6	0.270

a *First-degree relatives;*

b *E.g. antidepressants, asthma medication, insulin, levothyroxine and statins, contraceptives excluded;*

c *At least 30 minutes every week;*

d *Not significant after adjusting for concomitant type 1 diabetes. Data available in ≥97% of the patients on each variable except for working 188/237 and vitamin D supplementation 207/237*.

**Figure 2:**
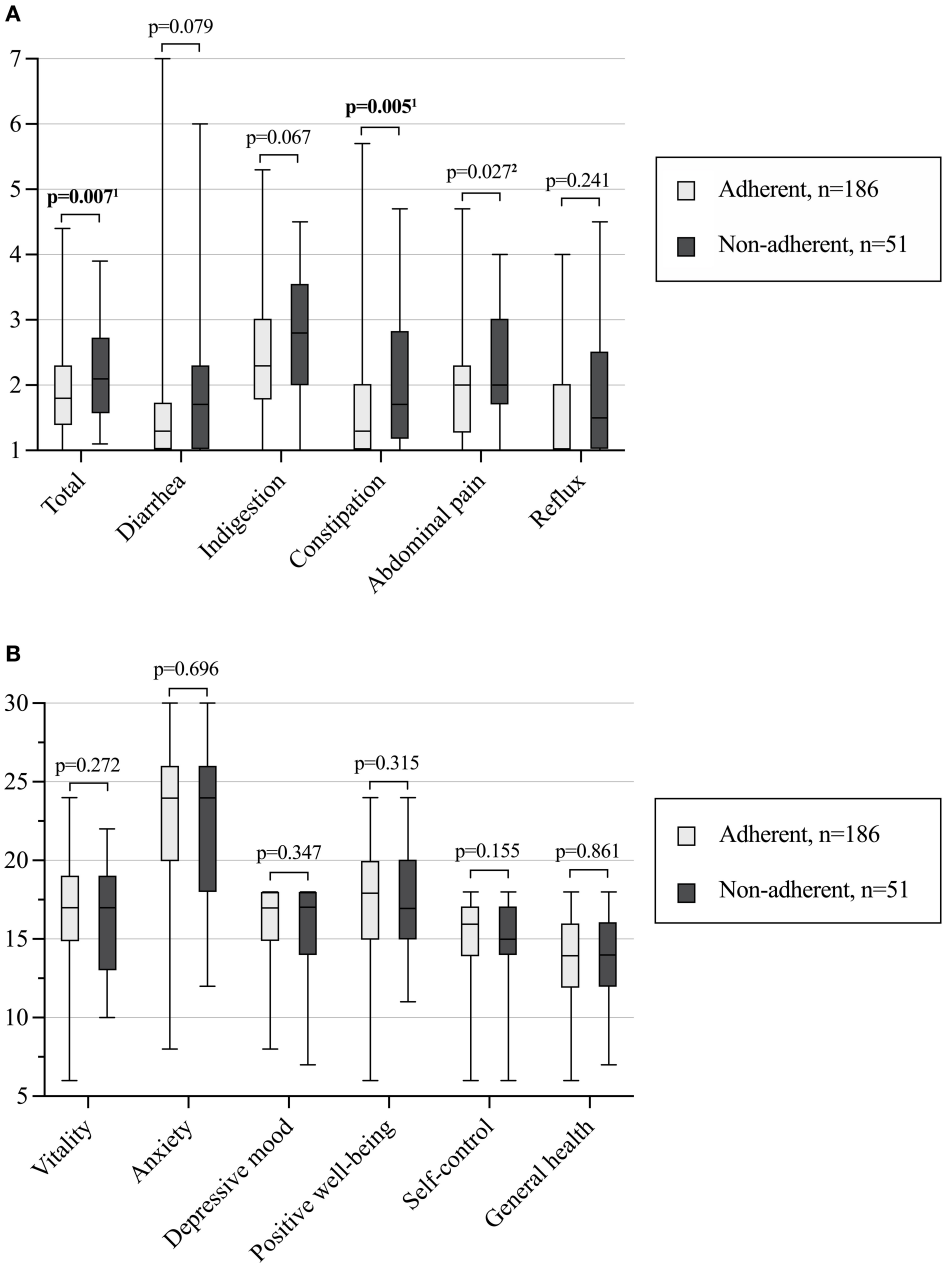
Median (horizontal line), interquartile range (box), and minimum and maximum values (vertical line) of current gastrointestinal symptoms **(A)** and health-related quality of life **(B)** in 237 adult celiac disease patients diagnosed in childhood and currently adherent or non-adherent to a gluten-free diet. Higher scores denote either more severe gastrointestinal symptoms **(A)** or better self-perceived quality of life **(B)**. ^1^Remained significant or ^2^not significant after adjusting with concomitant type 1 diabetes. Statistically significant *P*-values bolded.

Long-term non-adherent patients found gluten-free diet more difficult to maintain and more often reported celiac disease-related symptoms, whereas experiences or concerns about health, restrictions caused by the diet and presence of regular follow-up were comparable (Table 3). They had also more overall gastrointestinal symptoms, especially constipation and abdominal pain measured by the GSRS, while there were no differences in other sub-scores or in health-related quality of life measured by the PGWB (Figure 2). Adjustment for T1D did not affect the results despite abdominal pain, in which the difference was no longer significant.

**Table 3:** Current health, treatment experiences, and follow-up in 237 adult celiac disease patients diagnosed in childhood and currently adherent or non-adherent to gluten-free diet (GFD).

	**Adherent**, ***n*** **=** **186**		**Non-adherent**, ***n*** **=** **51**		
	**n**	**%**	**n**	**%**	**P-value**
Experienced health					0.056
Excellent or good	156	83.9	36	72.0	
Moderate or poor	30	16.1	14	28.0	
Concerns about health					0.081
None or minor	155	84.2	36	73.5	
Moderate or severe	29	15.8	13	26.5	
Symptoms related to celiac disease	35	19.2	19	38.8	**0.004** ^**a**^
Daily life restrictions due to GFD	85	45.9	23	50.0	0.622
Difficulties with the GFD					**0.001** ^**a**^
None or seldom	154	82.8	30	61.2	
Sometimes or often	32	17.2	19	38.8	
Follow-up of celiac disease					0.358
Regular	49	26.3	10	20.0	
None or occasional	137	73.7	40	80.0	

*Data was available for ≥95% cases in each variable.*

a *Remained significant after adjusting with concomitant type 1 diabetes. Statistically significant P-values presented in bold face*.

**Figure 3:**
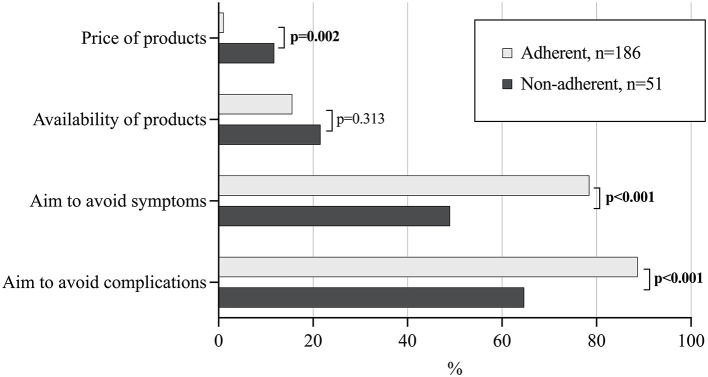
Significance of different patient- and diet-related factors for long-term dietary adherence in 237 adult celiac disease patients diagnosed in childhood and currently adherent or non-adherent to gluten-free diet. Statistically significant *P*-values presented in bold face, all remained significant after adjusting for concomitant type 1 diabetes.

The main decisive factors in both groups for long-term dietary adherence were attempts to avoid symptoms and complications, but these were considered less important and the price of gluten-free products more important among the non-adherent patients (Figure 3). Presence of T1D did not affect the significance of difference in these motivational factors. Specific explanations for ongoing or earlier non-adherence included being a teenager or student (*n* = 15), poor availability or quality of gluten-free products (*n* = 14), financial issues (*n* = 10), absence of gluten-induced symptoms (*n* = 6), difficulties in adhering to or failure to understand the diet (*n* = 3), anger or embarrassment due to the diet (*n* = 6), urge to eat gluten/interest in tasting normal food (*n* = 5), living abroad (*n* = 3), work-related eating situations (*n* = 1) and military service (*n* = 1).

In a subgroup analysis, adult celiac disease patients with concomitant T1D presented with poorer adherence to a gluten-free diet already in childhood follow-up, as well as reported poorer dietary adherence and more difficulties with the diet, more significant role of availability and price of gluten-free products for their adherence and more regular celiac disease follow-up in adulthood compared to those without T1D (Supplementary Table). The groups did not differ in current or diagnosis age, sex distribution, health experiences or prevalence of persistent symptoms (Supplementary Table).

## Discussion

One of our main findings was the generally good long-term adherence to a gluten-free diet (79% on a strict diet) among adult patients with a childhood diagnosis of celiac disease. Moreover, most patients reported occasional lapses and only 4% reported non-restricted gluten consumption or weekly lapses. So far a few studies with follow-up in adulthood of originally pediatric patients have reported variation in dietary adherence between 36–81% (12, 18–20), whereas those diagnosed in adulthood have shown slightly better long-term figures of 40–98% (19, 26–29). However, comparison between studies is not straightforward given the wide variation in study designs and characteristics of study populations, as well as in methods of measuring dietary adherence (30, 31).

We found long-term non-adherence to be overrepresented among patients with concomitant T1D. Earlier research findings on this issue are inconsistent (32–39), and the primary aim in most of these studies has been to evaluate the impact of gluten-free diet on diabetic control (34–37, 39) rather than to estimate dietary adherence as such (32, 33, 38). It is logical that the co-existence of two chronic conditions requiring continuous monitoring and care would increase the risk for non-adherence. However, these challenges may not emerge before responsibility for treatment shifts from caregivers to patients themselves in adolescence (33, 40), although here the difference was seen already during childhood follow-up. Also, availability and price of gluten-free products seemed to play especially significant role for those with coexistent T1D. In contrast to T1D, presence of a gastrointestinal comorbidity in the present cohort predicted better adherence. This may be attributable to attempts to exclude celiac disease-related symptoms with a strict diet before evaluations of possible co-existing conditions.

Although the non-adherent patients reported more ongoing symptoms, their experienced general health and quality of life were comparable to those who were strictly adherent. This could be partly explained by the observation that they were less often afraid of complications of untreated celiac disease. Ongoing gluten intake is the most common reason for symptom persistence after celiac disease diagnosis (41, 42) and avoidance of symptoms has previously been reported to motivate patients to maintain a strict diet, whereas the role of fear of complications is less clear (12). The relatively young age of our participants may have affected these experiences, as the possible negative long-term consequences of dietary lapses may have not yet have become apparent. Both ongoing symptoms and non-adherence have previously been associated with poorer quality of life, but whether there is a true causality remains unclear (43, 44).

Here, long-term non-adherent patients also found maintaining a gluten-free diet more challenging and reported that the price of products affected their adherence more often. In contrast, availability of products was considered less important, possibly reflecting the good availability in Finland. The high price of gluten-free foodstuffs has also previously been reported to predispose to non-adherence (26, 45, 46). Whether celiac disease patients receive financial support to cover the higher costs varies considerably worldwide (47). In Finland, children with confirmed celiac disease are currently entitled to monthly subsidies, whereas this is not the case among adults. Additional factors possibly explaining differences in the adherence include patients' knowledge about celiac disease and their self-confidence in managing the diet (26, 45, 48).

Excluding T1D, long-term difficulties with a gluten-free diet could not be predicted at celiac disease diagnosis or during childhood follow-up. Although most diabetics were diagnosed by risk-group screening, the baseline clinical features were not associated with adulthood dietary adherence. This is important, as there has been a fear that screening-detected patients might have poorer motivation to accept the diet than those detected due to symptoms (20, 49, 50). However, this was not supported by our findings. In theory, being diagnosed in childhood could promote adherence, as many of these patients have been on a gluten-free diet for almost their whole lives (18). Conversely, an early diagnosis could also predispose to reduced treatment motivation if the reasons for maintaining a restrictive diet have remained unclear (15, 51). Transition to adult care is a special phase when adolescents should assume responsibility for their treatment and cultivate skills for good treatment for the rest of their lives. Therefore, it would seem logical to support patients at that specific point, although more data is needed about the best practices for the transition process (52). In line with our earlier studies (15, 28), presence of adulthood follow-up or membership of a celiac society were not associated with dietary adherence here, whereas in some other countries both of these have been reported to have a positive effect (19, 53). External support could play a greater role if the general circumstances for a strict gluten-free diet are challenging. On the other hand, patients with concomitant T1D reported poorer dietary adherence despite more regular follow-up, suggesting that the physicians might have focused more on T1D during the visits.

### Strengths and Limitations

The major strengths of our study are the large and well-defined cohort of pediatric celiac disease patients and comprehensive long-term follow-up data after transition to adult care. However, the only moderate response rate for the follow-up questionnaires could predispose to selection bias, particularly as there were fewer T1D patients among the responders. Self-reported dietary adherence and other long-term follow-up data can be considered a limitation, and comparison of our results to studies which have used other questionnaires to evaluate the adherence might not be straightforward (20, 26). On the other hand, the accuracy of theoretically less subjective methods to measure adherence, such as celiac disease serology and the recently introduced gluten immunogenic peptides have also been challenged (54, 55). Another limitation is that we did not have detailed information about the patients' income levels, which could also affect their adherence.

## Conclusions

Despite the generally good adulthood adherence to a long-term gluten-free diet among these originally pediatric celiac disease patients, particularly those with a co-existing T1D, persistent symptoms and financial issues were at risk for non-adherence and challenges with the diet. These individuals should receive special attention during follow-up, especially during the transition from pediatric to adult care, when the foundations of commitment to life-long treatment are laid. Recognizing the patients' personal motivation factors toward the diet could provide further support for optimized long-term treatment and health outcomes.

## Data Availability Statement

The datasets presented in this article are not readily available because, privacy or ethical restrictions. Requests to access the datasets should be directed to laura.kivela@tuni.

## Ethics Statement

The studies involving human participants were reviewed and approved by Regional Ethics Committee of Tampere University Hospital, Tampere, Finland. Written informed consent to participate in this study was provided by the participants or their legal guardian/next of kin.

## Author Contributions

LK, AE, HH, and KKu designed the study. LK and HH were responsible for statistical analyses. LK and KKu collected the data. LK, AE, and KKu drafted the manuscript and wrote the final version. MR, HH, and KKa reviewed the paper for important intellectual content. All authors interpreted the results, approved the final draft submitted, and agreed to be accountable for all aspects of the work.

## Funding

This study was supported by the Foundation for Pediatric Research, the Competitive State Research Financing of the Expert Area of Tampere University Hospital, the Päivikki and Sakari Sohlberg Foundation, the Maire Rossi Foundation, the Maud Kuistila Foundation, the Mary and Georg Ehrnrooth Foundation, the Paolo Foundation, the Emil Aaltonen Foundation, the Finnish Celiac Society, the Sigrid Juselius Foundation, and the Academy of Finland.

## Conflict of Interest

LK, KKa, and KKu have received personal lecture fees from the Finnish Coeliac Society outside the submitted work and serve as members of the advisory committee of the Finnish Coeliac Society. The remaining authors declare that the research was conducted in the absence of any commercial or financial relationships that could be construed as a potential conflict of interest.

## Publisher's Note

All claims expressed in this article are solely those of the authors and do not necessarily represent those of their affiliated organizations, or those of the publisher, the editors and the reviewers. Any product that may be evaluated in this article, or claim that may be made by its manufacturer, is not guaranteed or endorsed by the publisher.
