# Supplementary material for: Coexisting Type 1 Diabetes, Persistent Symptoms, and Financial Issues Associate With Poorer Adherence to a Gluten-Free Diet in Celiac Disease After Transition From Pediatrics to Adult Care
Source: Front Nutr. 2022 May 26;9:883220. doi: 10.3389/fnut.2022.883220 (PMC9200750; doi:10.3389/fnut.2022.883220)
Supplement: Supplementary file 1 [file Table_1.docx]

| **Supplementary Table.** Characteristics in 237 adult celiac disease (CD) patients diagnosed in childhood and with or without concomitant type 1 diabetes (T1D). | | | | | | | | | | | | |  |
| --- | --- | --- | --- | --- | --- | --- | --- | --- | --- | --- | --- | --- | --- |
|  | |  | **T1D, n=18** | |  | |  | **No T1D, n=219** | |  | |  | |
|  | | **Median** | | **Quartiles** | | **Median** | | | **Quartiles** | | **P value** | |  |
| Age at CD diagnosis, years | | 7.4 | | 4.7, 12.5 | | 9.8 | | | 5.3, 13.6 | | 0.383 | |  |
| Current age, years | | 26.6 | | 22.2, 37.7 | | 27.1 | | | 22.1, 37.5 | | 0.919 | |  |
|  | | **n** | | **%** | | **n** | | | **%** | |  | |  |
| Girls | | 10 | | 55.6 | | 154 | | | 70.3 | | 0.192 | |  |
| Childhood GFD adherence^1^ | |  | | | |  | | | | | **0.008** | |  |
|  | Strict diet | 7 | | 53.8 | | 160 | | | 88.4 | |  | |  |
|  | Occasional lapses | 6 | | 46.2 | | 19 | | | 10.5 | |  | |  |
|  | No diet | 0 | | 0 | | 2 | | | 1.1 | |  | |  |
| Adulthood GFD adherence | |  | |  | |  | | |  | | **0.003** | |  |
|  | Strict diet | 9 | | 50.0 | | 177 | | | 81.2 | |  | |  |
|  | Occasional lapses | 4 | | 22.2 | | 27 | | | 12.4 | |  | |  |
|  | No diet | 5 | | 27.8 | | 14 | | | 6.4 | |  | |  |
| Difficulties with the GFD | |  | |  | |  | | |  | | **0.029** | |  |
|  | None or seldom | 10 | | 55.6 | | 174 | | | 80.9 | |  | |  |
|  | Sometimes or often | 8 | | 44.4 | | 41 | | | 19.1 | |  | |  |
| Daily life restrictions due to GFD | | 9 | | 56.3 | | 99 | | | 46.0 | | 0.430 | |  |
| Significant for adherence | |  | |  | |  | | |  | |  | |  |
|  | To avoid symptoms | 13 | | 72.2 | | 158 | | | 72.1 | | 0.994 | |  |
|  | To avoid complications | 15 | | 83.3 | | 183 | | | 83.6 | | 1.000 | |  |
|  | Availability of products | 9 | | 50.0 | | 31 | | | 14.2 | | **0.001** | |  |
|  | Price of products | 4 | | 22.2 | | 4 | | | 1.8 | | **0.001** | |  |
| Follow-up of CD | |  | |  | |  | | |  | | **0.001** | |  |
|  | Regular | 11 | | 61.1 | | 48 | | | 22.0 | |  | |  |
|  | None or occasional | 7 | | 38.9 | | 170 | | | 78.0 | |  | |  |
| Experienced health | |  | |  | |  | | |  | | 0.752 | |  |
|  | Excellent or good | 14 | | 77.8 | | 178 | | | 81.7 | |  | |  |
|  | Moderate or poor | 4 | | 22.2 | | 40 | | | 18.3 | |  | |  |
| Concerns about health | |  | |  | |  | | |  | | 0.749 | |  |
|  | None or minor | 14 | | 77.8 | | 177 | | | 82.3 | |  | |  |
|  | Moderate or severe | 4 | | 22.2 | | 38 | | | 17.7 | |  | |  |
| Symptoms related to CD | | 4 | | 22.2 | | 46 | | | 22.0 | | 1.000 | |  |
| ^1^6-24 months after the CD diagnosis. GFD, gluten-free diet. | | | | | | | | | | | | |  |
